# Fatigue Life Assessment of Welded Joints by Combined Measurements Using DIC and XRD

**DOI:** 10.3390/ma14195802

**Published:** 2021-10-04

**Authors:** Yixun Wang, Kazushi Ueda, Ryota Nagao, Seiichiro Tsutsumi

**Affiliations:** 1 Department of Joining Design and Dependability, Joining and Welding Research Institute, Osaka University, Osaka 567-0047, Japan; 2 Energy Solution & Marine Engineering Company, Kawasaki Heavy Industries, Ltd., Kobe 650-8670, Japan; ueda_kazushi@khi.co.jp; 3 Steel Research Laboratory, JFE Steel Corporation, Chiba City 260-0835, Japan; ry-nagao@jfe-steel.co.jp

**Keywords:** fatigue life, digital image correlation (DIC), X-ray diffraction (XRD), hammer peening, assessment method

## Abstract

The existing methods of assessing the fatigue life of welded joints fail to consider local strain ranges and mean stress at the weld toe. The present work proposes a novel approach to assessing the fatigue life of welded joints by conducting measurements with digital image correlation (DIC) and X-ray diffraction (XRD) in combination. Local strain ranges at the weld toe of gusset welded joints were measured by DIC. Hammer peening was conducted on the welded joints to introduce different initial stresses. The influence of mean stress was investigated by considering initial residual stress measured by XRD and a perfect plastic material model. The fatigue experiment was carried out on specimens with and without hammer peening. The results showed that hammer peening could offset adverse welding deformation effectively, and introduce significant residual compressive stress. The fatigue failure life increased by more than 15 times due to hammer peening. The fatigue initiation life assessed by the proposed method was close to that based on nominal stress, indicating that the proposed method is reliable for predicting the fatigue initiation life of welded joints.

## 1. Introduction

The out-of-plane gusset has been widely used in steel bridges to provide additional stiffness and to relieve local deformation induced by vehicle loadings [1,2,3]. The out-of-plane gusset is usually joined to steel bridge components by welding, which is prone to fatigue damage because of inevitable local residual stress, stress concentration at the weld toe or root, welding imperfections and increasing traffic volumes [4,5]. To decrease residual stress, or to reduce the local stress concentration of the weld, maintenance methods such as hammer peening [6], shot peening [7], grinding [8] and additional weld [9], etc., are often applied during the construction of steel components or when steel structures are in service. As the bead profile and local stress conditions of welded joints have been changed due to the application of maintenance methods, it is necessary to assess fatigue life according to variation in stress concentration and mean stress.

The fatigue initiation life of welded joints can usually be assessed based on stress ranges or strain ranges, namely, the stress method and the strain method. The existing stress method includes nominal stress [10], hot spot stress [11] and effective notch stress [12], indicating sustained efforts to approximate a real local stress state. The disadvantage of assessing fatigue life by stress method is that it is difficult to calculate the local stress at the weld toe or weld root. Effective notch stress is usually calculated using only the elastic behavior of the material [13,14], thus it may overestimate real local stress if the yield stress has been achieved. Similarly, nominal stress and hot spot stress do not address the local stress concentration in welded joints induced by variation in the bead profile [15]. The strain method confronts the same problem as the stress method, as the strain of a weld toe or weld root with a complicated profile is difficult to measure by conventional methods (such as the strain gauge) [16]. Another issue to be addressed is the measurement of mean stress [17]. Variation in mean stress at the weld toe or weld root can be induced by welding residual stress, applied stress ratio and bead profile. The influence of mean stress on fatigue life is usually assessed based on the nominal stress ratio [18], while mean stress at the weld toe can be quite different depending on the material plasticity and bead profile. Above all, the existing stress method and strain method have the disadvantages of failing to address material plasticity or measurement difficulties, which can result in an inaccurate fatigue life assessment.

The invention of digital image correlation (DIC) provides a new approach for measuring the strain of weld beads with complicated profiles [19]. The full-field strain distribution including structural discontinuities can be obtained during the fatigue loading using DIC. Ren et al. [20] used DIC to measure the strain history of butt-welded joints under low cycle fatigue. The fatigue life was predicted based on the Manson–Coffin formula, and showed favorable precision compared to the experiment results. Zheng et al. [21] assessed the fatigue life of NiTi alloy using nominal strain and local strain measured by DIC. The results indicated the non-trivial dependence of the fatigue life on the nominal strain variation, and the authors suggested to propose a material fatigue criterion based on the local strain variation. DIC can also be used together with optics and laser techniques for strain measurement. Corigliano et al. [22] proposed the thermographic method to predict the fatigue life of marine welded joints using a combination of DIC and infrared camera (IR). The above-mentioned studies worked on specimens of base metal or butt-welded joints with surface grinding and with residual stress eliminated. Welded structures can have more complex stress state and profile at the weld toe, and it is necessary to determine the initial stress and mean stress in order to achieve a precise prediction of fatigue life based on the measured strain ranges.

The novelty of this work includes the proposal of a fatigue life assessment method using combined measurements of DIC and X-ray diffraction (XRD), which solves the disadvantages of conventional assessments methods using stress ranges or strain ranges. DIC was used to measure local strain ranges of the weld toe. The mean stress was obtained by XRD measurement and the assumption of a perfect plasticity model. The specimens were also treated by hammer peening to achieve different initial stress states. The fatigue life was finally assessed by the proposed method and compared to the conventional methods and experiment results. It is expected that the present research would help make accurate predictions of fatigue life and serve as a reference in structural design.

## 2. Measurement of Residual Stress by XRD

### 2.1. The Hammer Peening Treatment

**Figure 1:**
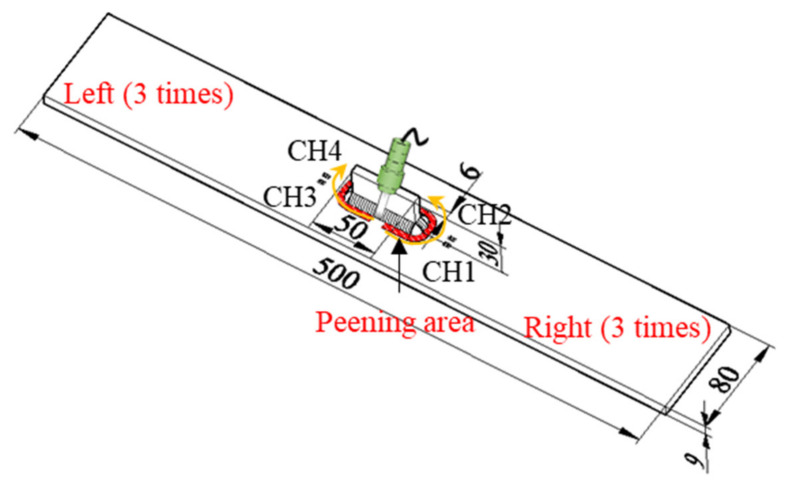
The specimen geometry (mm).

**Figure 2:**
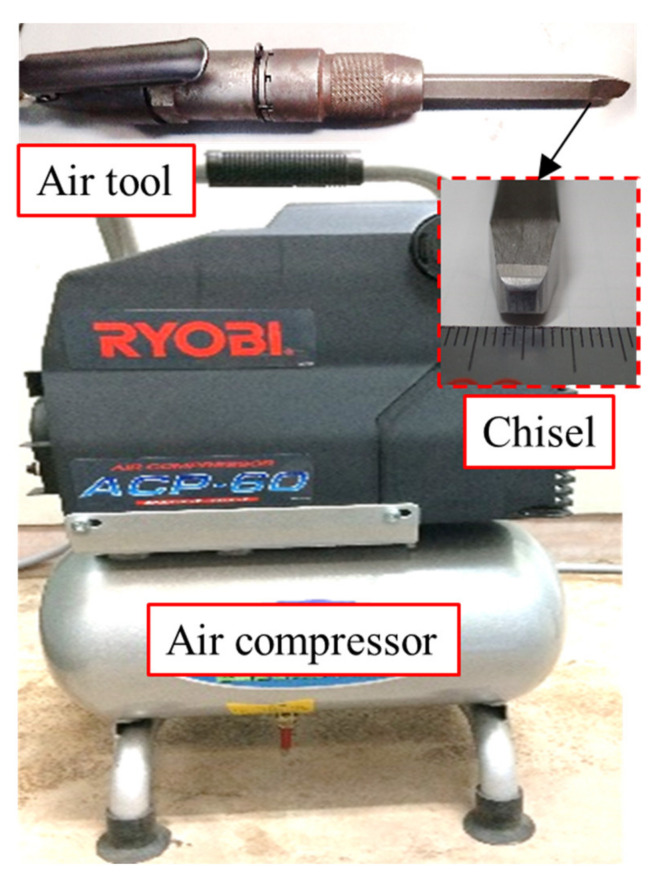
The hammer peening equipment.

**Figure 3:**
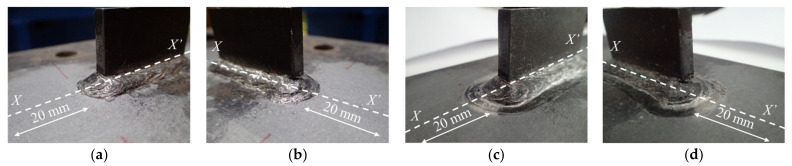
The bead profile: (**a**) Before hammer peening (*X*); (**b**) Before hammer peening (*X*’); (**c**) After hammer peening (*X*); (**d**) After hammer peening (*X*’).

Two specimens of out-of-plane gusset welded joints were fabricated using high strength steel HT780 with yield stress greater than 685 MPa [23], and joined by CO_2_ gas metal arc welding. The geometric dimension of the specimens is shown in Figure 1. To achieve a different level of initial stress, one of the specimens was treated by hammer peening. The treated specimen was fixed to the reaction frame by clamps in order to avoid possible displacement during the process of hammer peening. The devices to be used for hammer peening included a portable air compressor, an impact air tool, and a chisel as shown in Figure 2. The chisel tip was burnished to a flat rectangular surface of 4 mm × 5 mm with its corners rounded, and the operating frequency of the impact air tool was 90 Hz. The hammer peening was carried out from the welding end and operated along the side-weld for a range of 20 mm. The same process was conducted on the weld at least three times to ensure the maximum residual compressive stress was greater than 500 MPa [24]. The as-welded specimen was named by AW and that treated by hammering peening was named by HP. The bead shape of the specimen HP before and after hammer peening is shown in Figure 3. It could be observed that the transition from weld to base metal was much smoother after the hammer peening and the radius of the weld toe visibly increased. 

### 2.2. Residual Stress

**Figure 4:**
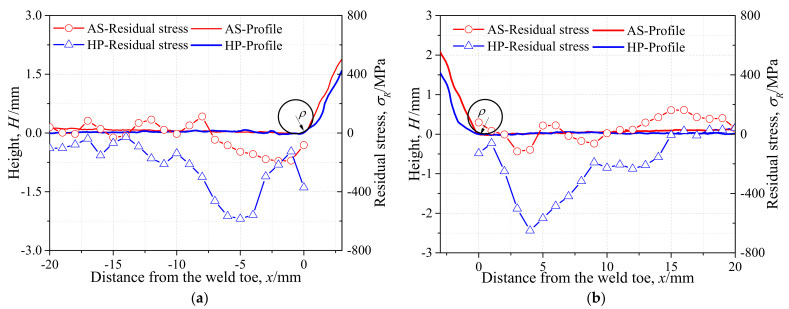
Angular distortion and residual stress in central axis *X*-*X*’: (**a**) *X* side; (**b**) *X*’ side.

The geometric deformation due to the welding and hammer peening was measured along the central axis *X*-*X*’ of the specimens, as shown in Figure 4. The original angular distortion due to the process of welding was 0.5°, and the application of hammer peening reduced the value to −0.1°; thus the hammer peening was able to effectively offset the adverse welding deformation. The radius of the weld toe was also measured for specimens AW and HP. The radius of the weld toe on side *X* increased from 1.5 to 2.2 mm, while the radius on side *X*’ increased from 1.2 to 2.5 mm due to the hammer peening. To determine the stress concentration factor (SCF) of the specimens, the finite element (FE) model was built based on the measured geometric dimensions. The static loading was applied on the FE model in the state of elasticity. The normal stress at the weld toe was divided by the nominal stress to obtain the SCF. The SCF of specimen AW was 2.78 and that of specimen HP was 2.06; thus, the stress concentration was greatly relieved at the weld toe by the hammer peening.

The XRD was applied to measure the residual stress of specimens AW and HP from the weld toe to the base metal along the deck surface, with a range of 20 mm every 1 mm, as shown in Figure 3. The residual stress distribution is illustrated in Figure 4. It was observed that the residual stress of specimen AW ranged from −200 to 160 MPa, while residual compressive stress was measured for all testing points in specimen HP. The welding residual tensile stress was obviously reduced by hammer peening, as the maximum compressive stress reached more than −600 MPa. The residual compressive stress reached its maximum at approximately 4 mm from the weld toe, which was the edge of the chisel used for hammer peening.

## 3. Measurement of Strain by DIC

### 3.1. Setup of the Experiment

**Figure 5:**
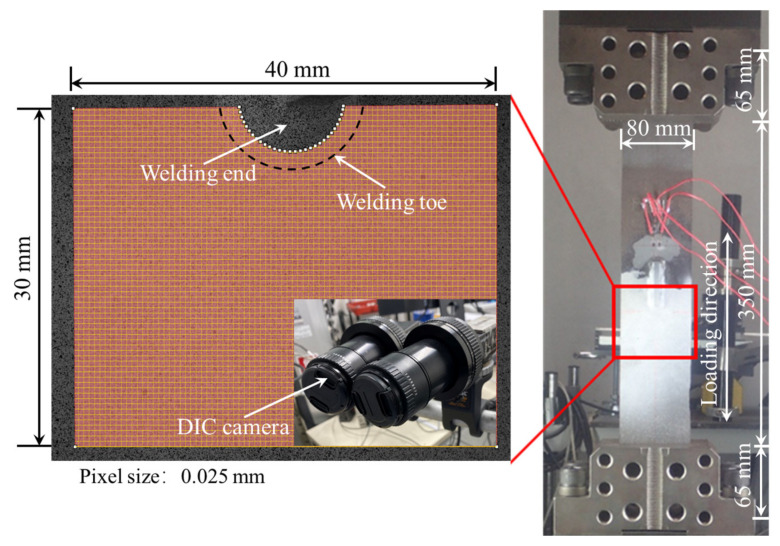
The DIC setup and loading conditions.

The fatigue loading was conducted on the specimens AW and HP, with strain measured by DIC. The nominal stress range Δ*σ* was 250 MPa, the stress ratio *R* was 0 and the loading frequency was 5 Hz. The fatigue loading was carried out by the fatigue machine through the clamps, which constrained both sides of the specimens. The constraint area and loading direction were indicated in Figure 5. DIC was used to measure the strain distribution of the specimen in the first 1000 cycles. A spatial resolution of approximately 6.25 × 10^−4^ mm^2^/pixel within the measurement area of 40 mm × 30 mm was achieved. The identical pixels before and after deformation were identified so as to calculate the displacement and strain based on the spatial relationship calibrated before the experiment.

Before the fatigue loading, the strain variation due to the clamping of the specimen was measured to obtain the initial deformation state. The lower side of the specimen was first constrained by the clamp and the surface deformation was recorded by the DIC cameras before and after the upper side of the specimen was fixed; thus, the initial strain *ε* due to the angular distortion when gripped by the clamps was measured, as shown in Table 1 and Table 2 (*n* = 0). It was observed that the initial strain on specimen AW (360 *με*) was much greater than that of specimen HP (−30 *με*) at *n* = 0, which indicated that the angular distortion of specimen HP due to welding was reduced by hammer peening. After the first 1000 cycles of fatigue loading, four strain gauges named by CH1-CH4 were attached 12 mm from the weld toe, as shown in Figure 1, to measure the nominal strain variation. The cyclic loading with the same loading parameters was continued until fatigue failure.

### 3.2. Strain Distribution in the First 1000 Cycles

Table 1 and Table 2 illustrate the strain contour of two specimens on side *X* under maximum and minimum loading when cycles *n* = 1, 3, 10, 100 and 1000. The peak strain was usually located at the weld toe of the gusset welded joint. The yield strain calculated by the design yield stress (685 MPa) was 3325 *με*, thus the material at the weld toe of specimen AW had yielded at the first cycle, while that of specimen HP remained in an elastic state when *n* ≤ 1000. For the specimen AW, *ε* under maximum and minimum loading tended to increase with the increase in loading cycles, because the yield stress of steel was reached and plastic deformation was accumulated due to the ratcheting effects. The change in strain range was small, ranging from 2900 *με* to 3150 *με*. For specimen HP, peak strain *ε_max_* remained unchanged generally with the increase in loading cycles, indicating that the steel was in a state of elasticity after the hammer peening. The strain range was stable and ranged from 2250 *με* to 2460 *με*, which was much smaller than specimen AW. Additionally, the peak strain *ε_max_* of specimen HP was usually approximately half that of specimen AW; thus, local stress in the vicinity of the weld toe was greatly reduced due to the effects of hammer peening.

**Figure 6:**
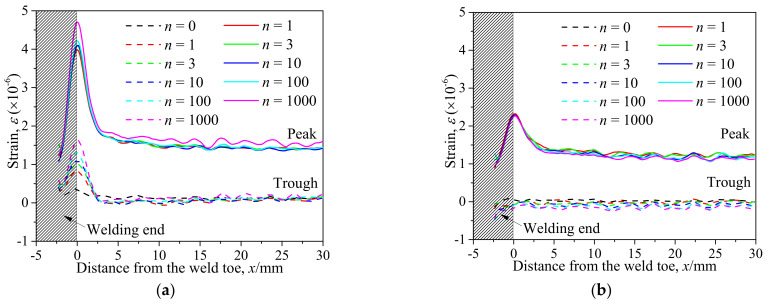
Strain distribution along the central axis *X-X*’: (**a**) Specimen AW; (**b**) Specimen HP.

Strain distribution along the central axis *X-X*’ of the specimens is shown in Figure 6. The bead is marked by the shadowed area. It was clear that the strain level at the weld toe was the greatest for both specimens due to the local stress concentration. The strain dropped dramatically from the weld toe and tended to plateau at a distance of 5 mm from the weld toe. Defining nominal strain by the ratio of nominal stress *σ* to elasticity modulus *E*, the peak value of nominal strain was approximately 1214 *με*. However, the measured peak nominal strain of specimen AW at *x* = 30 mm as shown in Figure 6a was 1464 *με*, approximately 250 *με* greater than the theoretic calculation. The difference most likely resulted from the initial strain during the clamping due to the angular distortion. The measured nominal strain of specimen HP (1241 *με*) as seen in Figure 6a was generally equal to the theoretic calculation because the angular distortion was offset effectively by the hammer peening, and the initial strain caused by the clamping was small.

## 4. Fatigue Life Assessed by Combined Measurements of DIC and XRD

### 4.1. Proposal of the Assessment Method

**Figure 7:**
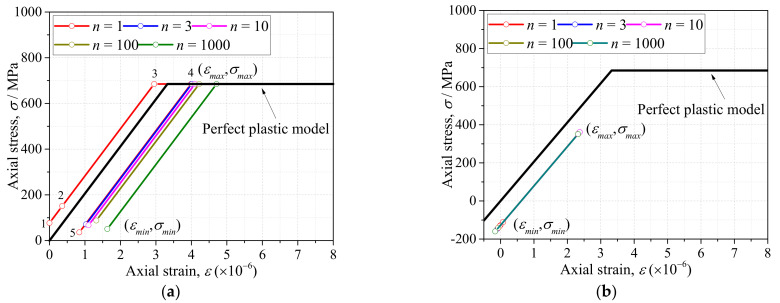
The stress-strain relationship of weld toe by perfect plastic model: (**a**) Specimen AW; (**b**) Specimen HP.

As described in the introduction, the existing strain method to assess the fatigue life of welded joints fails to consider the strain ranges of weld toes with irregular profiles, and the influence of mean stress is usually considered by nominal stress instead of local stress. The purpose of the proposed assessment method was to use DIC to measure the local strain ranges of the weld toe, while mean stress was calculated considering initial stress measured by XRD and a perfect plastic model. First, the strain ranges Δ*ε* of specimen AW and HP at the weld toe were measured by DIC as seen in Table 1 and Table 2. No obvious change of strain ranges Δ*ε* were observed from *n* = 1 to *n* = 1000 for specimens AW and HP, thus the stain ranges Δ*ε* at *n* = 1000 were used for fatigue life assessment. Second, the cyclic stress-strain (S-S) relationship at *n* = 1, 3, 10, 100, 1000 was calculated considering a perfect plastic model. The perfect plastic model was featured by an elasticity modulus *E* = 206 GPa, and yield stress *σ_y_* = 685 MPa. The loading cycle *n* = 1 was depicted by five stages as seen in Figure 7a, which illustrates the S-S relationship of specimen AW. The initial stress was defined by the residual stress (77 MPa) measured by XRD. The initial strain was defined by 0 because no loading was conducted (Stage 1). After the specimen was clamped at Stage 2, the measured axial stain at the weld toe was 360 *με*, and the corresponding stress calculated by the perfect plastic model was 151 MPa. As the fatigue loading increased, the material at the weld toe yielded at Stage 3, and the maximum strain *ε_max_* accumulated to 3990 *με* as measured by DIC at Stage 4. The peak stress *σ_max_* at *n* = 1 was determined to be 685 MPa, based on the material model. Subsequently, the unloading began, and the minimum strain *ε_min_* at *n* = 1 was measured by DIC (840 *με*), by which the corresponding *σ_min_* (36 MPa) could be calculated at Stage 5. The mean stress of the weld toe at *n* = 1 was therefore calculated by (*σ_min_* + *σ_max_*)/2, which was 360.5 MPa. The mean stress *σ_m_* of the weld toe at *n* = 3, 10, 100, and 1000 were obtained by the same method, and the cyclic S-S curves were illustrated in Figure 7a. The ratcheting strain could be easily observed in the first 1000 cycles of specimen AW and the *σ_m_* at *n* = 1000 was 367.76 MPa.

The S-S curves of specimen HP were obtained in Figure 7b by the same method as specimen AW, as was the mean stress at the weld toe. It was observed that the weld toe of specimen HP was consistently in an elastic state in the first 1000 cycles, which resulted from the significant residual compressive stress induced by hammer peening. The calculated mean stress *σ_m_* at *n* = 1000 was 95.48 MPa. Finally, the fatigue initiation life of specimens AW and HP was assessed by an improved Coffin–Manson equation considering influence of mean stress *σ_m_* as shown in Equation (1) based on the previous studies [25].

(1) $$f(\sigma_{m})\times \Delta \varepsilon =0.83N_{c}{}^{-0.606}+AN_{c}{}^{B}$$

where

(2) $$A=\frac{2.85711\times 10^{-3}}{{10,000}^{B}}$$

(3) $$B{=\log}_{200}\left(\frac{C+3.23306\times 10^{-3}}{2.85711\times 10^{-3}}\right)$$

(4) $$C=-{\left({\left(-1.95212\times 10^{-6}\sigma_{y}+2.93632\times 10^{-3}\right)}^{-30}+{\left(1.67957\times 10^{-3}\right)}^{-30}\right)}^{-\frac{1}{30}}$$

(5) $$f(\sigma_{m})=\frac{1}{1-4.1\times 10^{-4}\sigma_{m}-1.6\times 10^{-7}\sigma_{m}{}^{2}}$$

### 4.2. Assessment of Fatigue Life

**Figure 8:**
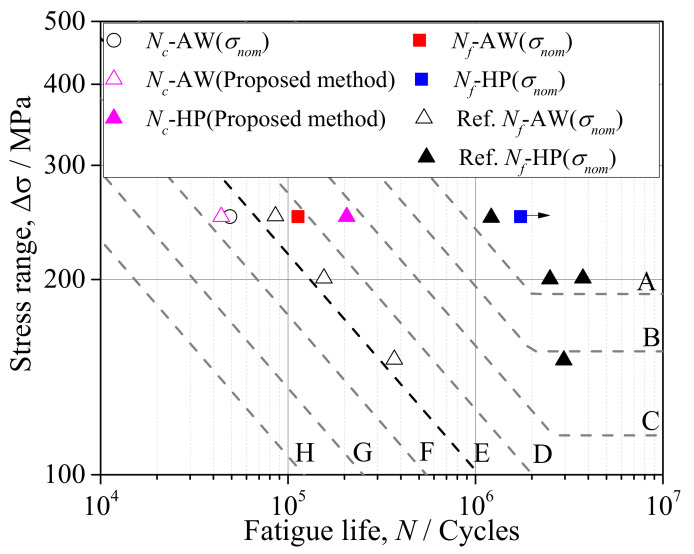
Fatigue life assessed by the proposed method.

The strain range Δ*ε* and mean stress *σ_m_* at *n* = 1000 were used for the prediction of crack initiation life by the proposed method, as the strain range Δ*ε* was stable when *n* was large enough. Additionally, the crack initiation life was represented by the nominal stress (*σ_nom_*) for comparison. The fatigue life of crack initiation measured by strain gauges was defined when the average value of gauge CH1 and CH2 or CH3 and CH4 (Figure 1) was reduced by 5%. The fatigue life of crack initiation (*N_c_*) and fatigue failure (*N_f_*) of the specimens AW and HP was assessed by *S-N* curves of typical fatigue details from Japan Society of Steel Construction (JSSC) [26] and shown in Figure 8. The studied out-of-plane gusset welded joints corresponded to Class E in the design specification of JSSC, and the testing data from previous studies marked by triangular symbols were also added for reference [27].

For specimen AW, the crack was initiated from the weld toe on the *X* side. The *N_c_* assessed by the proposed method was 44,025 cycles, which was slightly smaller than that represented by the nominal stress (49,000 cycles). The difference may result from the different definitions of crack initiation. It may nevertheless be concluded that the proposed method was reliable for assessing crack initiation life. The fatigue failure of specimen AW occurred at approximately loading cycle 113,000. The fatigue life of crack initiation accounted for approximately 39% of that of the fatigue failure. The data points from the reference [27] were also roughly scattered on the design curve of Class E.

For specimen HP, the *N_c_* predicted by the proposed method was approximately 205,792. However, the crack initiation did occur on the weld joint; the fracture occurred at the base metal clamped by the loading jig at approximately loading cycle 1,700,000. Therefore, the fatigue life of the out-of-plane gusset welded joint increased by more than 15 times after hammer peening. The fatigue life of specimens subjected to hammer peening in a previous study [27] also drastically increased, and it was observed that the fatigue strength of this detail was enhanced from class E to class A by hammer peening if assessed by *S-N* curves proposed by JCCS.

## 5. Conclusions

In the present study, a novel method of assessing fatigue life was proposed using the combined measurement of DIC and XRD. The basic principle of the proposed method was to assess fatigue life by the local strain range, considering the effects of local mean stress. The local strain range Δ*ε* at the weld toe was measured by DIC. The local residual stress *σ_R_* was measured by XRD and considered as the initial stress, so that the local mean stress *σ_m_* could be calculated by the variation of local strain considering a perfect plastic model. Fatigue life was finally assessed by an improved Coffin–Manson equation. The following conclusions were drawn:

(1) The original angular distortion due to the process of welding was 0.5° and the application of hammer peening reduced the value to −0.1°, indicating that hammer peening can effectively offset adverse welding deformation.
(2) The fatigue initiation life assessed by the proposed method was close to that represented by the nominal stress; thus, the proposed method is reliable for predicting the fatigue initiation life of welded joints.
(3) The hammer peening resulted in a decrease in SCF from 2.78 to 2.06, and the maximum compressive stress was as great as −600 MPa. Fatigue failure life increased by more than 15 times after hammer peening in this research.

## Figures and Tables

**Table 1:** Strain distribution measured by the proposed method (Specimen AW).

Cycles	*n* = 0 (After Clamping)		*n* = 1	*n* = 3	*n* = 10	*n* = 100	*n* = 1000
*ε_max_*	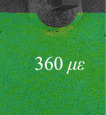		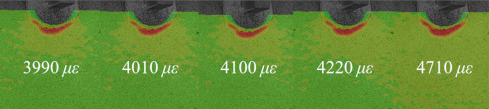				
*ε_min_*			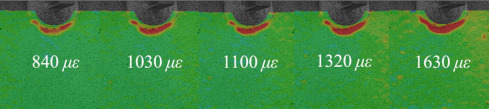				
Δ*ε*			3150 *με*	2980 *με*	3000 *με*	2900 *με*	3080 *με*

**Table 2:** Strain distribution measured by the proposed method (Specimen HP).

Cycles	*n* = 0 (After Clamping)		*n* = 1	*n* = 3	*n* = 10	*n* = 100	*n* = 1000
*ε_max_*	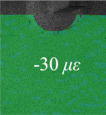		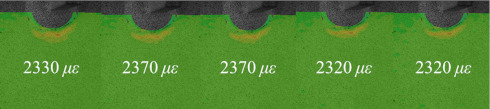				
*ε_min_*			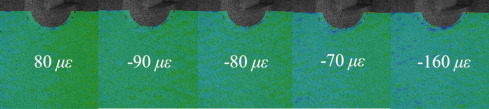				
Δ*ε*			2250 *με*	2460 *με*	2450 *με*	2390 *με*	2480 *με*

## Data Availability

The data presented in this study are available on request from the corresponding author. The data are not publicly available due to the data also form part of an ongoing study.
